# Does the socioeconomic context explain both mortality and income inequality? Prospective register-based study of Norwegian regions

**DOI:** 10.1186/1475-9276-10-7

**Published:** 2011-02-03

**Authors:** Jon Ivar Elstad

**Affiliations:** 1 Norwegian Social Research (NOVA), Oslo, Norway; 2 Department of Sociology and Human Geography, University of Oslo, Oslo, Norway

## Abstract

**Background:**

Studies from various countries have observed worse population health in geographical areas with more income inequality. The psychosocial interpretation of this association is that large income disparities are harmful to health because they generate relative deprivation and undermine social cohesion. An alternative explanation contends that the association between income inequality and ill health arises because the underlying social and economic structures will influence both the level of illness and disease and the size of income differences. This paper examines whether the observed association between mortality and income inequality in Norwegian regions can be accounted for by the socioeconomic characteristics of the regions.

**Methods:**

Norwegian register data covering the entire population were utilised. An extensive set of contextual and individual predictors were included in multilevel Poisson regression analyses of mortality 1994-2003 among 1.6 millions individuals born 1929-63, distributed across 35 residential regions.

**Results:**

Mean income, composition of economic branches, and percentage highly educated in the regions were clearly connected to the level of income inequality. These social and economic characteristics of the regions were also markedly related to regional mortality levels, after adjustment for population composition, i.e., the individual-level variables. Moreover, regional mortality was significantly higher in regions with larger income disparities. The regions' social and economic structure did not, however, account for the association between regional income inequality and mortality. A distinct independent effect of income inequality on mortality remained after adjustment for regional-level social and economic characteristics.

**Conclusions:**

The results indicate that the broader socioeconomic context in Norwegian regions has a substantial impact both on mortality and on the level of income disparities. However, the results also suggest, in a way compatible with the psychosocial interpretation, that on top of the general socioeconomic influences, a higher level of income inequality adds independently to higher mortality levels.

**Previous publication:**

This article is a reworked version of the study 'Er inntektsforskjeller dødelige?' [Are income inequalities lethal?] which was published in Norwegian in *Tidsskrift for velferdsforskning *[Journal for welfare research], Vol. 13 (4), 2010.

## Background

The "big idea" [1] that in contemporary affluent countries, large income disparities are in themselves detrimental for health, has triggered theoretical and methodological discussions [2-7] as well as many empirical studies, see reviews in [8-13]. Results have been diverse and sometimes conflicting. Nonetheless, a considerable number of empirical studies have observed an inverse relationship between population health and the level of income inequality, both in ecological and multi-level designs, after adjustment for confounders of different types. Recent examples include both between-country studies [14,15] and within-country studies [16-20].

How to explain this association is however disputed. The psychosocial interpretation [2,4,7,21-23] argues that among the fairly well-situated majority in contemporary rich countries, economic and material circumstances do not directly translate into health. Rather, a psychosocial mediation takes place. Unequal distributions of material resources become reflected in people's perceptions of their social standing, in their feelings of relative deprivation, and in unsatisfactory experiences of social interactions and social cohesion. In turn, these perceptions and experiences affect health because they contribute to feelings of insecurity and inferiority and induce unhealthy behaviour and stress-related biological reactions which become embodied as disease risk. Through such processes, higher levels of income inequality will eventually result in higher levels of ill health and mortality.

Various evidence suggests that such processes may be operating [2,24-26], but findings are mixed, e.g., [27,28], and the sheer complexity of the psychosocial theory is a challenge for empirical research. Also other explanations for the relationship between income inequality and health have been proposed. For instance, the association could arise simply because of the curvilinear relationship between individual income and health risk [29], but studies suggest that not much of the association can be explained in this way [30,31]. The neo-material interpretation claims on the other hand that the psychosocial theory greatly exaggerates the role of psychological mechanisms in present-day societies [9,32]. Rather, the relationship between contextual income inequality and ill health emerges because those placed at the lower end of the income scale will, in inegalitarian societies, have particular difficulties in obtaining the health-beneficial goods which are typical of modern society, such as well-equipped dwellings, safe cars, recreation facilities, and high-quality medical services. The neo-material explanation emphasises in particular that political regimes which restrict excessive income inequalities will also be more likely to provide social investments which enhance health, such as primary health services, schools, and regulations of work conditions.

A different explanatory approach is to follow the causal chain backwards and ask whether underlying economic and social structures create both the patterns of income inequality and produce ill health [6,33]. Social class structures, property relations and the composition of economic branches, the politics of trade unions, technological change, the interplay between state regulation and market mechanisms, and other societal aspects will influence income distributions. Such factors could also generate social circumstances with important health effects. This approach suggests therefore that the level of income inequality should be regarded as an outcome of a variety of social and economic forces and conditions which also, through other causal chains, could be influencing how population health develops. Even if a correspondence between income inequality and ill health is observed, the causal chain from income inequality via intermediary factor to population health could actually be quite weak, but this will easily be overlooked if research narrowly searches for pathways from income inequality to health without considering the overall socioeconomic context.

### Purpose and model

In this paper, the latter explanatory alternative is in focus. We utilise data from nation-wide population registers in order to examine the interrelationship between socioeconomic context, income inequality and mortality in Norwegian regions. Together with Sweden, Finland, and Denmark, Norway belongs to the Nordic, social-democratic type of welfare states [34] which, among other things, is characterised by comparatively small income inequalities [35]. Finnish, Swedish and Danish studies have found few links between contextual income inequality and indicators of population health (see [36-40]). It has therefore been suggested that in egalitarian countries, variations in income inequality is practically unrelated to health outcomes [2,9,13,41]. Norway seems to be a deviating case, as multi-level analyses utilising population registers with extensive adjustment for individual characteristics have found a significant tendency to higher mortality risk in the more inegalitarian Norwegian municipalities and regions [16,17]. However, these studies have not provided much insight into how the association between mortality and income inequality has emerged, and it is uncertain how psychosocial mediations or other processes are involved.

**Figure 1:**
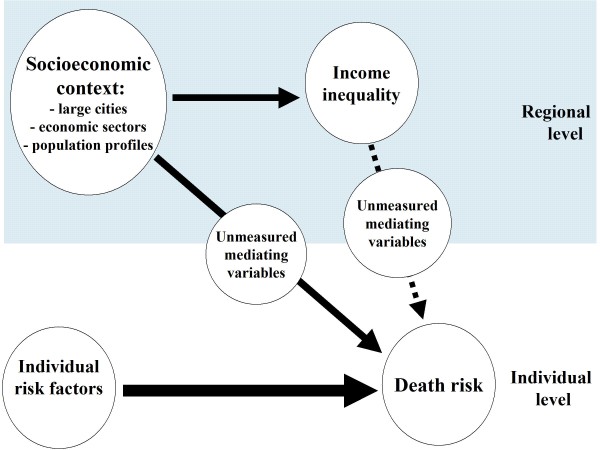
**Model**.

Figure 1 illustrates the model applied in this study. We assume that individual risk-factors, both biological, social and economic, are major predictors of all-cause mortality, but variations in individual-level characteristics (i.e., differences in the composition of regional populations) will not account for all the regional differences in mortality. The model suggests that various aspects of what can broadly be termed the socioeconomic context of the region - the overall income level, the level of urbanisation, the composition of economic sectors, and population profiles - also influence the level of mortality in the region, on top of the effects of individual-level characteristics. This might occur through various pathways. A higher overall income level could imply better public services. The larger cities might induce unhealthy ways of living [42]. Career and work stress, as well as crime, drug abuse, and prostitution, may be especially prevalent among certain sections of the urban population. The composition of economic sectors can have health implications both because of typical work conditions in different industries and because the employees of particular branches (e.g., factory workers) have distinct health-related lifestyles. Also plausible is that not only individuals' own educational level, but even the predominant educational level in the area, can have health effects. If highly educated people are numerous in the surrounding area, their more healthy lifestyles might spread in the population more rapidly than if the highly educated constitute a small minority [43]. The proportion of disability benefit recipients varies considerably between Norwegian geographical areas, and their higher death rates could possibly lead to higher mortality rates in areas where they are numerous.

The model also suggests that the regional socioeconomic context will influence, or at least correspond to, variations in income inequality. Selective migration may play a part, as both certain sections of the poor (homeless, drug addicts, perhaps some immigrant categories) as well as the especially affluent may seek to live in central urban areas, which may lead to larger income disparities. Furthermore, the composition of economic branches will probably be related to the level of income inequality. The higher salaries among employees in the financial services may lead to larger income differentials, while income inequalities may be smaller if large population sections are factory workers with fairly homogeneous wages or farmers with little variation in holding size. How educational levels are distributed in the population may also affect the income distribution - there will probably be less income inequality if only a minority of the population have higher education, but more income inequality if higher education is more widespread, because this could imply that the population is broadly distributed across different educational levels.

Accordingly, a plausible hypothesis is that the underlying socioeconomic context will correspond to the level of income inequality and will, at the same time, be implicated in health-generating processes. A possibility is therefore that the association between regional mortality and income inequality which has been observed in Norway, could, fully or partially, be accounted for in this way. To explore this is the main purpose of the present study.

### Design considerations

Stated otherwise, this paper aims at examining two types of place effects on mortality in conjunction: Effects arising from the broader socioeconomic context as described above, and effects linked to the level of income inequality, which could, possibly, be mediated by the mechanisms proposed by the psychosocial theory. This purpose requires choices as to how analyses should be performed, and design questions are discussed in this section.

First, the topic presupposes that people are assigned to places, i.e., to locations in geographical space. On what criteria the area classification should be done, depend on the purpose of the study and what kind of social processes one wants to highlight [44,45]. Proponents of the psychosocial interpretation have argued that psychosocially mediated health effects will seldom arise because of the level of income inequality in the neighbourhood or local community [2,11]. It is contended that the proliferation of mass media in recent times, television in particular, has transferred people's point of reference, important for how relative deprivation and other psychosocial mechanisms arise, from the immediate social surroundings to larger geographical areas such as regions, states (in the US), or even countries. A practical application of this idea, utilised in this paper, is to construct a limited number of relatively large geographical entities which approximate "societies" in terms of sharing, for instance, a labour market, trade and administrative centres, local newspapers, and ways of talking (dialects). As Norway's population was only about 4.5 millions in the 1990s, a "large" Norwegian region may appear relatively small in international comparisons.

A second design consideration relates to migration. Estimation of place effects on health becomes complex if a substantial number of people migrate between areas and are exposed to different area contexts during the study period [44]. Moreover, possible place effects on an individual's risk of mortality will seldom appear immediately after moving into an area, but rather arise little by little from experiencing the social context over time. If migration is selective for health-related factors, further estimation problems arise [46,47]. In order to diminish such problems, the study sample in this paper has been restricted to "permanent residents", i.e., those living in the same region during the entire period 1992-2002 - or, for those who deceased, from 1992 until death.

The focus on permanent residents enhances the likelihood that the study sample has been exposed to the same regional context during a number of years. A related question concerns the stability of the area exposures themselves. If the contextual characteristics change, questions arise as to what contextual circumstances the population actually has been exposed to. Most of the contextual factors utilised in this paper refer to conditions which do not change rapidly, such as the composition of economic sectors and the level of urbanisation. A particular topic is however the stability of income disparities over time. This has implications for the psychosocial theory, which proposes that income inequality is an important cause for relative deprivation and social cohesion. Such facets of social life will probably emerge gradually, and they must have some duration in order to produce health effects. Therefore, it can be argued that the mechanisms the psychosocial theory highlights, presuppose considerable stability in contextual income disparities. If income inequality fluctuates drastically from year to year, the relationship between income inequality and enduring features of social life will be difficult to determine (although, of course, frequent changes in income inequality might themselves influence social life). This problem is however of little relevance for the present study. As income information for each year 1993-2002 was available, we could establish that the level of income inequality in each region, as well as the rank order of the regions on the scale of income inequalities, had remained remarkably stable during this period (Pearson's r between regions' Gini coefficients for 1993 and 2002 was 0.96). This reflects the stability in the Norwegian economy during these years. In the analyses, 1993 income information is utilised for calculating income inequality, but this measurement will in practice indicate levels of inequality for the entire 1990s.

In line with common practice [13,35], Gini coefficients which vary from 0.00 (all incomes equal) to 1.00 (one person/unit receives all the income), are used for estimating levels of income inequality. When examining whether income inequality has consequences for the social environment, one must however ask not only how inequality should be calculated, but also whose incomes are relevant. For instance, in regions with many students and retired elderly with low incomes, Gini coefficients can be inflated, but if the retired are marginal in community life and students mostly temporary inhabitants, their incomes might have less impact on social life than the incomes of long-term inhabitants in working age. For this reason, calculations of income inequality are based only on the incomes of permanent residents age 30-64. Furthermore, it can also be discussed what income definition is appropriate for the present topic. While the total pre-tax income of an individual may be particularly relevant for a person's prestige and social status, his/her household-adjusted post-tax income will better reflect his/her consumption level. These two types of incomes are probably associated, but will they influence social interaction in the same way? In this paper, analyses are performed with regional parameters calculated both from pre-tax personal income and from household-adjusted post-tax income.

## Methods

### Data and area classification

Data are constructed by Statistics Norway's linkages of individual information from administrative registers [48], which has been anonymised for research purposes. The registers cover the entire Norwegian population, and missing values are negligible. This paper analyses men and women born 1929-1963 who were alive at the end of 1993 (when aged 30-64) and either died 1994-2003 or were still living in Norway at the end of 2003.

The area definition builds on Statistics Norway's application of the European Community's NUTS4 standard for economic regions [49], which seeks to delimit areas which share labour markets, trade centres, etc. The original classification divides Norway into 90 economic regions, but many of them are small. In view of the above discussion about area size, smaller neighbouring economic regions have been collapsed into larger adjacent areas, while the large capital region Oslo has been divided between the rich West and the less affluent Eastern part. Thus, the study population was assigned to 35 areas, termed regions in this paper.

In the material, there were 1,805,496 men and women born 1929-1963 with vital information up to 2003. Among them, 10.0 per cent changed residential region 1992-2002 and were therefore excluded from the analyses. Also a few thousand persons with negative incomes were excluded, because the analyses presuppose adjustment for individuals' incomes, but persons with negative personal income are difficult to place in the income hierarchy. Otherwise, missing information was very scant. The sample with information on all individual-level variables used in the analyses numbered 1,621,202 persons.

### Individual and regional variables

The mortality data 1994-2003 had information about year and month of death, allowing for analyses which take the time of death into account. In addition to sex and age, individual variables include education per 1992, marital status in January 1993, immigrant status (defined as first-generation immigrant without Norwegian background), disability benefit recipient per December 1993, and total pre-tax personal income in 1993, classified into eight categories. Pre-tax personal income is the sum of salaries and wages, net income from own business (relevant for farmers, self-employed, etc.), pensions and public benefits (such as pre-retirement pension, disability pension, unemployment benefits), income from capital, and a few other minor sources.

Two dummy variables were used to indicate urbanicity: Living in Oslo (the capital with about 0.5 million inhabitants, the only large city in Norway), and residing in one of the medium-sized cities Stavanger, Bergen or Trondheim (with populations about 90,000 to 150,000 during the 1990s). The composition of economic branches was indicated by three variables: Percentage of the employed population working in agriculture, forestry or fishing, percentage in manufacturing and mining, and percentage in banking and financial services. This information was gathered from the 1990 Census. More detailed economic branch information is available, but some trials indicated that these three variables capture fairly well the structural differences between regions. Regional variables indicating the percentage in the population aged 30-64 with university or college education, and the percentage receiving disability benefits, were also calculated from the material.

Variables indicating regions' average income level and income inequality (Ginis) were calculated, as said above, from the 1993 incomes of permanent residents, aged 30-64, both from personal pre-tax income and from post-tax household-adjusted income. The latter was calculated by summarising the post-tax income of all family members and dividing the sum with the square root of the number of family members. For these estimations, negative incomes were excluded, while those with incomes above 10 millions Norwegian Kroner (NOK) were recoded to 10 M in order to avoid unreasonably influence by a few "super-rich".

Table 1 describes the data. During 1994-2003, about 80,000 deaths occurred, with a total exposure time of about 15.8 millions person-years. The mean population in the regions (age 30-64 in 1993) was about 46,000. To illustrate the size of mortality difference between the regions, age- and sex-adjusted death rates (deaths per 100,000 person-years) were calculated, showing a range from 449 to 795 around a mean of 550. The composition of economic branches, as well as the prevalence of higher education and especially the prevalence of disability pensioners, varied considerably between the regions. The mean personal pre-tax income in the regions was about 200,000 NOK (Norwegian Kroner), clearly higher than the average household-adjusted post-tax income which was about 171,000 NOK. Using the personal pre-tax income definition, Ginis varied between the regions from 0.27 to 0.43 (mean 0.32). When calculated from household-adjusted post-tax income, Ginis were, as could be expected because of progressive taxation, considerably lower (range 0.19-0.36, mean 0.22).

**Table 1:** Descriptive statistics

*Individual-level variables (N = 1,621,202)*	*Percent*		
Men	50.2		
Women	49.8		
			
Married in 1993	70.3		
Never married	15.5		
Previously married	14.2		
			
Immigrant, first generation, no Norwegian background	4.1		
Recipient of disability pension 1993	10.7		
			
University, higher level	11.1		
University, lower level, college	10.1		
Secondary education, higher level	23.0		
Secondary education, lower level	28.9		
Basic education	27.0		
			
Pre-tax personal income, 1993			
- 400,000 Norwegian Kroner (NOK) +	5.3		
- 300,000 - 399,000 NOK	8.1		
- 250,000 - 299,000 NOK	11.0		
- 200,000 - 249,000 NOK	20.0		
- 150,000 - 199,000 NOK	21.5		
- 100,000 - 149,000 NOK	16.4		
- 50,000 - 99,000 NOK	10.9		
- 0 - 49,000 NOK	6.9		
			
Age in 1993 - mean (SD)	45.5 (9.6)		
			
Number of deaths 1994 - 2003	80,653		
Number of person-years 1994 - 2003	15,845,468		
			
*Contextual variables (N = 35 regions)*	*Mean*	*SD*	*Min - max*
Sample, aged 30-64, permanent residents	46,300	22,550	19,400 - 129,000
Deaths 1994-2003 per 100 000 person years^a^	550.4	68.6	449 - 795
			
Percent of total employment in 1990, main branches			
- agriculture, forestry, fishing (%)	7.2	5.0	0.4 - 18.8
- manufacturing, mining (%)	17.3	4.8	9.4 - 26.7
- banking, financial services (%)	6.7	3.6	3.0 - 16.6
			
University/college education, age 30-64 (%)	20.0	7.8	12.5 - 50.2
Disability pension recipients, age 30-64 (%)	11.1	2.7	5.9 - 16.9
			
Mean pre-tax personal income 1993, age 30-64 NOK	199,500	26,400	174,300-302,800
Mean household-adjusted post-tax income 1993 age 30-64	171,300	16,400	156,200-235,700
			
Ginis, pre-tax personal income, age 30-64	0.318	0.033	0.274 - 0.434
Ginis, household-adjusted post-tax income 1993, age 30-64	0.216	0.031	0.118 - 0.363

^a^Men and women together, adjusted for sex and age according to European standard population.

### Statistical analyses

The analyses will examine deaths in a sample of some 1.6 million individuals who are nested within higher-level units, i.e., 35 regions. This requires a multi-level technique [10,50], and the very large sample and a dichotomous outcome (dead/not dead 1994-2003) make Poisson regression suitable [37,51]. The sample was aggregated into cells representing all combinations of residential region, sex, age (seven age categories), education (five categories), personal income (eight categories) and the other individual-level variables (see Table 1). In principle, this could result in more than 200,000 cells, but since many cells were empty, the actual number of cells turned out to be 92,752. The exposure in terms of person-years and the number of deaths were summarised for each cell. The counts of deaths are assumed to approximate a Poisson distribution. Regional variables constitute covariates. Estimations were made with the Stata program xtpoisson [52,53]. Random intercept models were fitted, i.e., it is assumed that the effects of individual and regional characteristics are basically similar across all regions, while the intercept (suggesting the overall mortality level in the region) varies, and the analyses indicate, among other things, to what extent individual-level and contextual-level variables can account for the intercept variation. IRR - incidence relative risk - is reported, with interpretation analogous to odds ratios. Predictors are recoded to facilitate interpretation; thus, the unit for Ginis is 0.05 point, while the unit for the proportion with higher education is 5 percentage points. Because the focus is on possible place effects on mortality, sex is used as a control variable and gender-specific results are not presented.

## Results

The correlation matrix of regional-level variables (Table 2) shows that Ginis estimated from pre-tax personal income and from post-tax household-adjusted income are closely related: Pearson's r = 0.88. Moreover, the matrix reveals a typical pattern: At the regional level, economic and social characteristics tend to have substantial co-variation. Regional Ginis are strongly associated with regional mean income - correlation coefficients are in the range 0.75 - 0.86. The percentage of highly educated people are strongly and positively related to income inequality, income level, and percentage employed in banking and financial services, but negatively related to percentage employed in agriculture etc. and percentage receiving disability benefits. Regional income inequality is lower the more employment in agriculture etc., but higher the more employment in banking and finance.

**Table 2:** Correlation matrix (Pearson's r), regional variables (N = 35)a

	2	3	4	5	6	7	8	9
1. Gini pre-tax personal income	.882	.789	.745	-.583	(.115)	.640	.755	-.495
2. Gini post-tax household-adj. income		.859	.840	-.543	(-.231)	.728	.855	-.437
3. Mean pre-tax personal income			.982	-.566	(-.310)	.822	.955	-.706
4. Mean post-tax household-adjusted income				-.509	(-.334)	.756	.921	-.657
5. Percentage employment agriculture, forestry etc.					(.115)	-.741	-.605	(.199)
6. Percentage employment manufacturing, mining						-.448	(-.399)	(.085)
7. Percentage employment banking, financial services							.851	-.578
8. Percentage aged 30-64 with university or college education								-.628
9. Percentage of population 30-64 with disability benefits								

^a ^All correlations, except those bracketed, are statistically significant, p < .01.

These strong inter-correlations make it difficult to disentangle the effects of the different contextual aspects from each other. Contextual characteristics cluster in many ways. For instance, a large banking/finance sector will usually correspond to higher overall income levels because of higher salaries, but therefore also to wider income disparities. Moreover, it will coincide with urbanisation and therefore go together with a contraction of agricultural employment and a growth in the proportion highly educated in the population.

Nonetheless, Table 2 suggests, in line with Figure 1, how the level of income inequality in a region reflects a variety of economic and socioeconomic conditions. To the extent that the correlations represent causal processes (which is debatable), the causal direction seems obvious. The level of income inequality can hardly be the cause of the economic structure or the overall educational level, but rather an outcome of processes linked to the economy and the profiles of the population. How strongly the level of income inequality depends on this overall socioeconomic context is illustrated by a multiple OLS regression analysis with the 35 regions as units. Together, the variables for mean regional income, economic structure, proportion with higher education, and proportion disability recipients account for more than three quarters of the variance in Ginis based on pre-tax personal income (adjusted R^2 ^= 0.782, table not shown).

Results from four random-intercept multi-level Poisson regression analyses are shown in Table 3. All analyses are adjusted for age (a categorical variable indicating seven 5-year bands) - the age effects, which follow familiar patterns, are not shown for space reasons. In these models, Ginis and mean regional income are calculated from pre-tax personal income.

**Table 3:** Multilevel Poisson regression of deaths 1994-2003,^a ^four random intercept models.

	Model 1		Model 2		Model 3		Model 4	
	IRR	95%CI	IRR	95%CI	IRR	95%CI	IRR	95%CI
*Individual-level predictors*								
Women (reference men)	0.460	0.452;0.467	0.459	0.452;0.467	0.459	0.452;0.467	0.459	0.452;0.467
								
Married (ref)	1.00		1.00		1.00		1.00	
Not married/never	1.692	1.658;1.726	1.692	1.658;1.727	1.691	1.658;1.726	1.692	1.658;1.727
Previously married	1.604	1.576;1.633	1.604	1.576;1.632	1.603	1.575;1.631	1.603	1.575;1.632
								
Immigrant	0.855	0.822;0.889	0.855	0.824;0.890	0.853	0.821;0.887	0.853	0.820;0.887
Disability pension	2.086	2.048;2.124	2.086	2.050;2.125	2.085	2.048;2.123	2.085	2.048;2.123
								
University, higher (ref)	1.00		1.00		1.00		1.00	
University, lower	1.182	1.135;1.231	1.182	1.136;1.231	1.182	1.135;1.231	1.182	1.135;1.231
Secondary, higher	1.241	1.197;1.286	1.242	1.198;1.286	1.242	1.198;1.286	1.241	1.198;1.286
Secondary, lower	1.297	1.253;1.344	1.298	1.254;1.345	1.299	1.254;1.345	1.299	1.254;1.345
Basic education	1.438	1.389;1.489	1.440	1.390;1.491	1.440	1.390;1.491	1.440	1.390;1.491
								
400,000 NOK+ (ref)	1.00		1.00		1.00		1.00	
300-399,000	1.100	1.048;1.155	1.101	1.049;1.156	1.101	1.049;1.158	1.101	1.049;1.156
250-299,000	1.168	1.115;1.224	1.169	1.117;1.225	1.169	1.116;1.224	1.169	1.116;1.225
200-249,000	1.291	1.236;1.348	1.292	1.237;1.350	1.292	1.237;1.349	1.292	1.237;1.350
150-199,000	1.485	1.421;1.551	1.487	1.423;1.553	1.487	1.423;1.553	1.487	1.424;1.553
100-149,000	1.700	1.626;1.778	1.703	1.629;1.780	1.703	1.629;1.780	1.703	1.629;1.780
50-99,000	1.959	1.871;2.051	1.962	1.874;2.054	1.963	1.875;2.055	1.963	1.875;2.055
0-49,000	2.036	1.938;2.139	2.038	1.940;2.141	2.039	1.941;2.142	2.039	1.941;2.142
								
*Region-level predictors*								
Gini, unit 0.05			1.090	1.046;1.136			1.112	1.057;1.170
Mean income^c^					1.002	0.978;1.027	0.985	0.964;1.007
								
Not large/medium city					1.00		1.00	
Oslo (capital)					1.103	0.998;1.219	1.079	0.994;1.171
Medium-size cities					0.955	0.898;1.018	0.950	0.903;0.999
								
Employment, education, disability, unit 5 percentage points								
- agriculture, forestry etc.					0.968	0.933;1.005	0.965	0.936;0.995
- manufacturing, mining					1.005	0.980:1.031	0.966	0.939;0.994
- banking, finance					1.110	1.027;1.200	1.089	1.021;1.161
- with higher education					0.979	0.938;1.022	0.965	0.931;1.000
- disability recipients					1.034	0.973;1.099	1.002	0.951;1.056
								
-2LL	149049.5		149035.9		149003.1		148989.1	
Change -2LL vs Model 1			- 13.6		- 46.4		- 60.4	
Intercept Standard Deviation	0.09718	0.07601; 0.12425	0.07935	0.06185; 0.10180	0.04540	0.03370; 0.06116	0.03514	0.02524; 0.0483

Gini and mean region income based on pre-tax personal income. Age adjusted^b^

^a ^1,621,202 individuals age 30-64, aggregated to 92,752 cells, nested in 35 regions. Offset ln person-years.

^b ^Age adjusted, seven 5-year categories, age effects not reported.

^c ^Unit 10,000 NOK (Norwegian Kroner).

Model 1, Table 3, includes only the individual-level predictors. Death risk among women was less than half of men's risk, married had lower risk, and mortality increased clearly with lower education, lower personal income, and among disability pension recipients. Immigrants' mortality risk, after adjustments for other individual characteristics, was lower than in the majority population. Furthermore, it can be noted that when contextual predictors are included in Models 2-4, only minor changes in the individual-level coefficients occur.

The focus of this paper, however, is not the individual-level predictors, but the effects of the contextual characteristics. In Model 2, regional income inequality is included in addition to the individual-level predictors. The effect (IRR = 1.090) is clearly significant in statistical terms. Thus, per 0.05 unit increase in Gini, the increase in mortality risk, adjusted for individual-level characteristics, is estimated to be nearly 10 per cent. The overall model fit is significantly improved from Model 1: - 2LL is reduced by 13.6, with one degree of freedom (1 df). The intercept standard deviation was also somewhat reduced (from 0.097 to 0.079), but is still highly significant, implying that the regional variations in mortality are only partially accounted for by individual predictors plus income inequality.

In Model 3, Table 3, regional income inequality has been taken out of the model, while the other predictors of socioeconomic context have been included: overall income level, urbanicity, economic structure, and the variables for population profiles. Clearly, these predictors are relevant for accounting for mortality levels. The model fit was significantly improved (-2LL reduction from Model 1 is 46.4, with 8 df, p < .001) and the intercept standard deviation was more than halved from 0.097 in Model 1 to 0.045 in Model 3. These predictors are often highly interconnected (Table 2) and could for this reason be treated as a bloc indicating central aspects of the overall socioeconomic context. Only one of the predictors - percentage of population employed in banking and finance - reaches statistical significance in Model 3.

The crucial test of the model in Figure 1, however, is to what extent regional income inequality, on top of and in addition to the contextual predictors utilised in Model 3, contributes to the variations in regional mortality levels. Model 4 includes all the contextual predictors. It turns out that the effect of the regional Ginis remains fairly strong and statistically significant (IRR = 1.112). Accordingly, the contention that the effects of regional income inequality would more or less vanish when the underlying socioeconomic context is taken into account, is not supported. Rather, it seems that larger income disparities correspond to higher mortality levels, whatever the configuration of mean income level, urbanicity, economic structure and population profile. As to the effects of the other contextual variables, their inter-correlations make firm conclusions difficult. Tentatively, it can however be suggested that a tendency to lower mortality levels can be observed in regions with higher mean income, in medium-sized cities, in regions with much agriculture, and in regions with a higher level of education. On the other hand, regions with higher employment in banking and finance display a tendency to higher mortality, but the interpretation of this is not self-evident.

The analyses in Table 3 were performed with Ginis and mean income levels calculated from pre-tax personal incomes. To examine the robustness of these findings, all analyses were repeated with Ginis and mean income levels calculated from the more consumption-sensitive income definition post-tax household-adjusted income. Table 4 shows the relevant results (individual-level effects are practically identical to those shown in Table 3 and are not shown). In Model 4, with all contextual variables, the effect of income inequality is fairly strong (IRR = 1.138). Thus, this alternative way of estimating regions' mean income and income inequality confirms the picture given in Table 3 - not unexpected since the correlation between the two ways of calculating Ginis and mean income was high (cf. Table 2).

**Table 4:** Multilevel Poisson regression of deaths 1994-2003,^a ^random intercept models, effects of regional variables adjusted for individual-level variables (not reported).

	Model 2		Model 3		Model 4	
	IRR	95%CI	IRR	95%CI	IRR	95%CI
*Region-level predictors*						
Gini, unit 0.05	1.105	1.059;1.152			1.138	1.048;1.236
Mean income^b^			1.007	0.980;1.036	0.977	0.946;1.008
						
Not large/medium city			1.00		1.00	
Oslo (capital)			1.107	1.001;1.224	0.959	0.844;1.090
Medium-size cities			0.957	0.899;1.018	0.938	0.886;0.992
						
Employment, education, disability, unit 5 percentage points						
- agriculture, forestry etc.			0.968	0.933;1.004	0.964	0.933;0.997
- manufacturing, mining			1.006	0.981:1.031	0.987	0.962;1.012
- banking, finance			1.112	1.029;1.202	1.116	1.041;1.195
- with higher education			0.975	0.941;1.011	0.958	0.926;0.991
- disability recipients			1.035	0.971;1.098	0.999	0.943;1.057
						
-2LL	149032.9		149002.9		148994.4	
Intercept Standard Deviation	0.07573	0.05889; 0.09740	0.04520	0.03352; 0.06093	0.03905	0.02858; 0.05336

Gini and mean region income based on post-tax household-adjusted income

^a ^Sample 1,621,202 individuals age 30-64 (1993), grouped in 92,752 cells and 35 regions. Offset ln person-years. ^b ^Unit 10,000 NOK (Norwegian Kroner).

## Discussion

### Main findings and interpretation

These analyses indicate that regional mortality levels among adults in Norway varied not only with variations in individual-level characteristics, but also with what can broadly be termed the socioeconomic context, i.e., the overall income level, urban ways of living, the composition of economic sectors, and the proportion of highly educated in the population. Together, such contextual factors constitute place effects which, in addition to individual-level variables, account for a considerable part of regional differences in mortality levels.

In addition to this, however, the analyses indicate that the level of regional income inequality is independently associated with mortality. Larger income disparities go together with somewhat higher death risk in the regional population, also after a series of other contextual factors are taken into account. The purpose of this paper was to examine the hypothesis that the co-variation between income inequality and mortality, observed in Norway and other countries, emerges because the underlying socioeconomic context contributes to population health and influences, at the same time, the level of income inequality. These analyses certainly indicate that the broad socioeconomic context is influential on mortality levels. Nevertheless, the analyses also indicate that regional income inequality is not simply a proxy for the socioeconomic basis, because after taking this socioeconomic context into account, a clear mortality effect of income inequality remains. Thus, regional income inequality seems to be independently related to the mortality level across many configurations of regional overall income, economic structure, and population profiles.

The findings of the present study are therefore compatible both with a traditional understanding of the causes for population health and with an interpretation which points to specific health effects of levels of income inequality. The overall mortality effects of the broader socioeconomic context observed in this study are in line with well established theories about pathways to health and disease. The effects of the mean income level, the economic structure, and the percentage highly educated, for instance, could represent the impact of material environments, working conditions, and lifestyles. Nonetheless, the finding that the effect of income inequality remains across different economic structures and population profiles, suggests that higher income inequality influences the social environment in unhealthy ways, perhaps through a psychosocial mediation which involve more relative deprivation and less social cohesion. But, as indicated by Figure 1, data on such mediating mechanisms are not available, and in this paper we cannot examine more closely the various possible pathways between income inequality and mortality.

### Strengths and limitations

An obvious advantage of this study is data elicited from population registers which implies a very large and unbiased sample and measurements with high validity. It is very plausible that the statistical patterns displayed in this study is congruent with the actual situation in Norway during the years around the millenium. Thus, the main conclusion - that regional mortality levels are clearly influenced by the broader socioeconomic context, but in addition will higher levels of income inequality correspond to higher levels of mortality - seems quite firm.

Still, many questions remain. A general limitation with studies based on population registers is the restricted access to individual data. This limitation is also relevant here, as individuals' circumstances are only described by background information on age, education, etc., and, as mentioned above, to follow causal chains through intermediary variables is not possible. This also concerns the distinction between compositional (individual-related) and contextual effects. Measurements of individuals' life-course exposures to working conditions, unhealthy lifestyles, and stressful life events, are lacking, and some of the contextual effects observed in these data would perhaps disappear with better individual-level data. On the other hand, the distinction between individual-level and contextual-level effects is blurred in reality and effects are intermingled and can manifest themselves at several levels [44]. Further investigations of how the observed statistical patterns emerge would be interesting, for instance by means of qualitative studies of social life in strategically selected regions.

It can be asked whether design choices (discussed in the Design considerations subsection) have influenced results. The area classification was grounded in typical arguments for the psychosocial interpretation [11] and based on principles constructed for economy statistics [49], but we do not know whether other area classifications would have given other results. A related issue is why income inequality effects on mortality appear in Norway, but only sporadically in other Nordic welfare states. One cannot exclude the possibility that relevant social processes occur differently in Norway, but it can also be noted that the design of most other Nordic studies has been different from the present one, i.e., relying on survey data [36], or utilising smaller areas [37-39], or having restricted variation in contextual income inequality [37,40,51].

In this study, the analysed sample was restricted to permanent residents in working age, in order to avoid "contamination" from movers with weak ties to specific regions. Tests show, however, that results were similar when movers were included - not surprising as movers constituted only about 10 per cent of the data material. It can be noted, interestingly, that separate analyses of the movers fail to find any systematic associations with regional contexts, which indirectly give credibility to the findings of contextual effects in this study.

Multilevel regression analyses with dichotomous outcomes in such large materials are demanding for statistical software, and the tendency to multicollinearity among the contextual variables makes precise estimation of effects difficult. The analyses presented in Table 3 and Table 4 were also performed by the Stata program Gllamm [52], with practically identical results - or in some instances, with more distinct effects of some of the contextual variables, for instance regional income inequality and the effect of living in the large Oslo city.

## Conclusions

The main purpose of this paper has been to examine the hypothesis that the association between population mortality and the level of contextual income inequality, observed in Norway and other countries, is primarily due to how the underlying social and economic structure of an area influences both mortality risk and the size of income disparities. The analyses of 1.6 millions individuals aged 30-64 at baseline, distributed across 35 regions, show that 10-years mortality risk was not only related to individuals' characteristics, but also clearly influenced by the characteristics of the regions' economic structure and the socioeconomic profiles of the regional population. On top of that, larger income disparities tended to go together with elevated mortality risk. The results indicate that the broader socioeconomic context is markedly influential both on mortality and on the level of income disparities. However, the results also suggest, in a way compatible with the psychosocial interpretation, that apart from the influences of the general socioeconomic characteristics of an area, a higher level of income inequality adds independently to higher mortality levels.

## Competing interests

The author declares he has no competing interests.
